# Non-Additive Effects of Genotypic Diversity Increase Floral Abundance and Abundance of Floral Visitors

**DOI:** 10.1371/journal.pone.0008711

**Published:** 2010-01-14

**Authors:** Mark A. Genung, Jean-Philippe Lessard, Claire B. Brown, Windy A. Bunn, Melissa A. Cregger, Wm. Nicholas Reynolds, Emmi Felker-Quinn, Mary L. Stevenson, Amanda S. Hartley, Gregory M. Crutsinger, Jennifer A. Schweitzer, Joseph K. Bailey

**Affiliations:** Department of Ecology and Evolutionary Biology, University of Tennessee, Knoxville, Tennessee, United States of America; Trinity College Dublin, Ireland

## Abstract

**Background:**

In the emerging field of community and ecosystem genetics, genetic variation and diversity in dominant plant species have been shown to play fundamental roles in maintaining biodiversity and ecosystem function. However, the importance of intraspecific genetic variation and diversity to floral abundance and pollinator visitation has received little attention.

**Methodology/Principal Findings:**

Using an experimental common garden that manipulated genotypic diversity (the number of distinct genotypes per plot) of *Solidago altissima*, we document that genotypic diversity of a dominant plant can indirectly influence flower visitor abundance. Across two years, we found that 1) plant genotype explained 45% and 92% of the variation in flower visitor abundance in 2007 and 2008, respectively; and 2) plant genotypic diversity had a positive and non-additive effect on floral abundance and the abundance of flower visitors, as plots established with multiple genotypes produced 25% more flowers and received 45% more flower visits than would be expected under an additive model.

**Conclusions/Significance:**

These results provide evidence that declines in genotypic diversity may be an important but little considered factor for understanding plant-pollinator dynamics, with implications for the global decline in pollinators due to reduced plant diversity in both agricultural and natural ecosystems.

## Introduction

Plant genetic variation and genotypic diversity consistently affect community and ecosystem properties across systems and environments [see review by – 1]. Such effects of plant genetic variation in dominant plant species on biodiversity and ecosystem function have important basic and applied implications for restoration and consequences of climate change [2] and place community and ecosystem ecology in an evolutionary framework. Although most of the research to date has focused on the interaction of plant genetics and arthropod herbivores, the effects of genetic variation in dominant plant species may also be important to plant-pollinator interactions. However, there are little data on the role of plant genetic variation in the structure of pollinator communities or affecting pollinator visitation [3]. Understanding the consequences of intra-specific genetic variation and genotypic diversity within plant communities on the local diversity and abundance of insect pollinator communities is an important ecological frontier and there are many reasons why this represents a critical research direction to explore.

First, inter-specific plant diversity can have positive effects on insect pollinator diversity and flower visitation [4]–[7]. Similarly, plant genetic diversity may increase pollinator abundance and richness via facilitative interactions of neighboring plants and greater variation in floral forms and nectar quality leading to a higher diversity of flower visitors [4], [8]–[10]. Second, many phenotypic traits that affect pollinators are genetically controlled, including floral traits, floral abundance, and flowering phenology [11]–[12]. For example, Holtsford and Ellstrand (1992) found that genetic and environmental variation affected traits related to gender separation in both space (anther-stigma separation) and time (protandry) [12]. Third, across multiple plant systems, plant genetic variation can have strong effects on arthropod and microbial communities and ecosystem level processes such as productivity and nutrient cycles [see reviews by ]–[1,13–15]. For example, genotypic differences in *Populus* can account for up to 70% of the variation in microbial community composition [16]. Fourth, the effects of genotypic diversity and stand-level genetic variation on community and ecosystem phenotypes [17]–[26] can be three times as high as the “average ecological effect size” as estimated in Möller and Jennion's (2002) meta-analysis [1], [27]. Fifth, the effects of genotypic diversity are often non-additive and synergistic, indicating that interactions between neighboring genotypes can cause outcomes which differ from additive expectations [17]–[18], [23]–[25]. More evidence is emerging suggesting that when synergistic neighborhood effects occur, they are fundamental to the maintenance of biodiversity and ecosystem function [1], [6], [28]. However, little is known about the role of intraspecific genetic variation and diversity on pollinator visitation. Non-additive, synergistic models may provide a critical mechanism for understanding if diverse plant communities are more attractive to pollinators. Lastly, if genetic variation in plants affects pollinator communities, then evolutionary processes in plants will probably have extended consequences on pollinators and the ecosystem services they provide. Together these points suggest that intraspecific plant genetic factors may be fundamental to plant pollinator interactions and the ecosystem services that they provide.

In a two year study, using a common garden which manipulated *Solidago altissima* genotypic diversity, we hypothesized that: [1] plant genetic variation for flowering time influences the abundance of flower visitors, and [2] genotypic diversity influences floral abundance, flower visitor richness and flower visitor abundance visitation through synergistic effects between plant genotypes. Together these hypotheses suggest that genetic variation at the patch-level may facilitate non-additive effects that positively impact insect flower visitors.

## Results

### Genetic Variation

We found that *S. altissima* genotype explained 43–76% of the variation in floral abundance, 56–86% of the variation in flower visitor abundance, and 46–57% of the variation in flower visitor taxonomic richness (**Table 1**). Because the abundance of individuals is commonly correlated with the number of taxonomic groups present in a community it is important to control for the effects of abundance on richness to determine if plant genetic factors directly impact flower visitor richness. We conducted a second analysis in which plant genotype and flower visitor abundance were the independent variables and flower visitor richness was the dependent variable. In this model, only flower visitor abundance was related to flower visitor richness (Flower visitor abundance: F_(1,63)_ = 22.80, p<0.0001; Genotype: F_(20,63)_ = 1.09, p = 0.425), indicating that the effects of plant genotype on flower visitor richness were a consequence of plant traits that influence flower visitor abundance.

**Table 1:** Analysis of genetic variation for individual and community phenotypes.

Phenotype	Year	*X* ^2^_(1)_	p	*H* ^2^†
Floral Abundance	2007	9.71	0.0009	0.76±0.46
	2008	18.32	<0.0001	0.43±0.22
Flower Visitor Abundance	2007	131.11	<0.0001	0.56±0.34
	2008	118.31	<0.0001	0.86±0.57
Flower Visitor Richness	2007	70.27	<0.0001	0.46±0.18
	2008	45.64	<0.0001	0.57±0.31

Results are from restricted estimated maximum likelihood (REML) ratio tests. Data from single genotype plots were used to test genetic variation in individual and community phenotypes.

**Figure 1:**
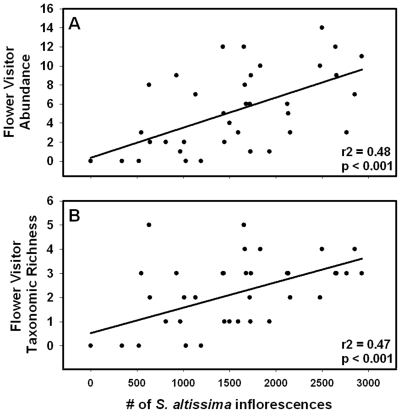
Relationships between floral abundance and flower visitors. Genetic relationships between floral abundance (number of inflorescences) and a) flower visitor abundance and b) flower visitor taxonomic richness (n = 42). Flower visitor abundance and taxonomic richness represent the total number of individuals and taxonomic groups observed per two minute time period. Each point represents a genotype mean (n = 2). Data represent observations from 2007.

We found significant phenotypic correlations between floral abundance of *S. altissima* plants and both flower visitor abundance and taxonomic richness (**Figure 1**, **Table 2**). To reduce the environmental contribution to a phenotype, using phenotypic correlations, we conducted genetic correlations [29], [30]. Consistent with the phenotypic correlations, we found similar patterns across years for genetic correlations between floral abundance and flower visitor abundance and richness (Table 2). This result indicates that plant genetic factors can impact the diversity of floral communities through genotypic variation for floral abundance. Because there was significant genetic variance for floral abundance and flower visitor community phenotypes, these results suggest that: 1) evolutionary processes that impact plant floral phenotypes may have consequences on associated interacting species; and 2) population level plant genotypic diversity might directly impact plant traits important to flower visitors, such as floral abundance, while indirectly affecting flower visitor abundance due to effects on plant traits.

**Table 2:** Phenotypic and genotypic correlations.

Phenotypic Correlations	Year	F_(1,42)_	p	r^2^
Floral Abundance and	2007	37.48	<0.0001	0.48
Fl. Visitor Abundance	2008	46.33	<0.0001	0.54
Floral Abundance and	2007	35.4	<0.0001	0.47
Fl. Visitor Richness	2008	44.82	<0.0001	0.53

Genotypic Correlations	Year	F_(1,21)_	p	r^2^
Floral Abundance and	2007	29.69	<0.0001	0.61
Fl. Visitor Abundance	2008	37.83	<0.0001	0.67
Floral Abundance and	2007	22.56	<0.0001	0.54
Fl. Visitor Richness	2008	33.48	<0.0001	0.64

Phenotypic and genotypic correlations between floral abundance (number of inflorescences) and flower visitor abundance and flower visitor taxonomic richness for the years 2007 and 2008. Phenotypic correlations are shown for all 42 single genotype plots, and individual genotypic correlations are shown using the mean value of all 21 genotypes.

### Genotypic Diversity

**Figure 2:**
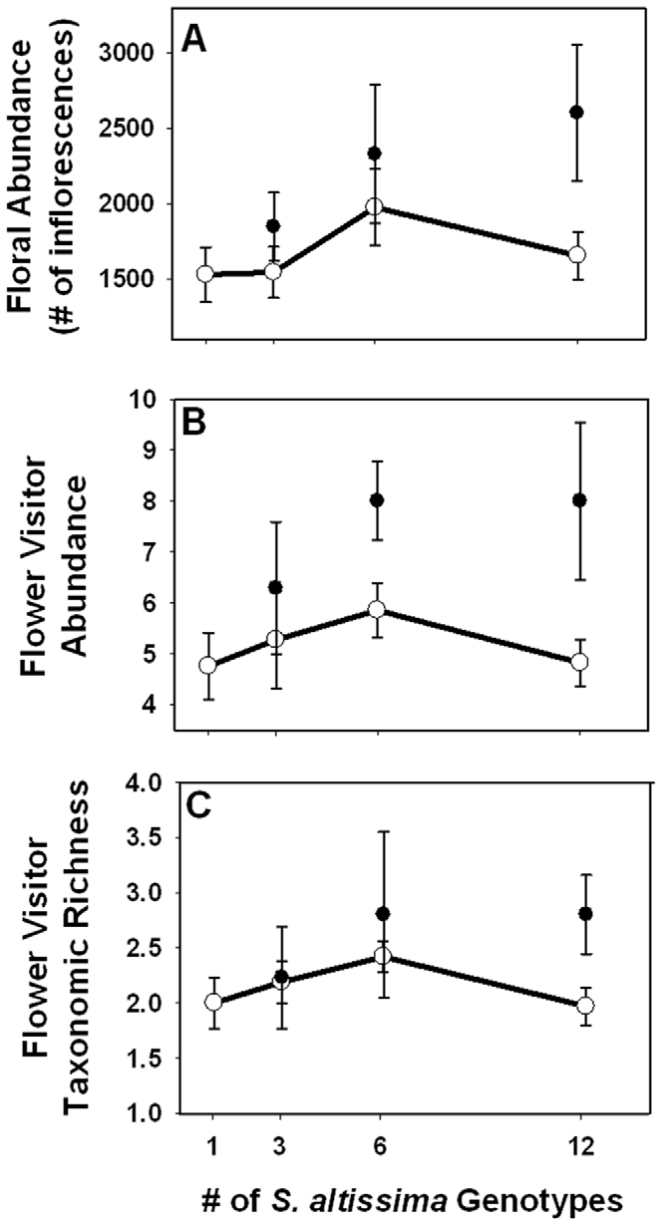
Genotypic diversity effects on floral abundance and flower visitors. Effects of genotypic diversity on a) floral abundance (number of inflorescences), b) flower visitor abundance and c) flower visitor taxonomic richness. The open symbols and error bars represent the mean and 95% confidence intervals results from the null model, based on re-sampling from the single genotype plot means. The filled symbols represent the observed mean values (±1 SE) for each diversity treatment. When the filled symbols fall outside the range of the null model 95% confidence interval, it indicates that the observed value for the diversity treatment is significantly different from additive expectations. Data represent observations from 2007.

Genotypic diversity also had a significant, positive impact on floral abundance in 2007 (**Figure 2a** - 2007: F_(1,63)_ = 7.68, p = 0.007, r^2^ = 0.11), flower visitor abundance in 2007 and 2008 (Figure 2b - 2007: F_(1,63)_ = 7.07, p = 0.010, r^2^ = 0.10; 2008: F_(1,63)_ = 5.49, p = 0.022, r^2^ = 0.08), and flower visitor richness in 2007 and 2008 (Figure 2c - 2007: F_(1,63)_ = 4.09, p = 0.048, r^2^ = 0.06; 2008: F_(1,63)_ = 11.99, p = 0.001, r^2^ = 0.16). Our data suggested a trend between genotypic diversity and floral abundance in 2008 (F_(1,63)_ = 2.90, p = 0.094, r^2^ = 0.05). We conducted a second analysis in which genotypic diversity and flower visitor abundance were the independent variables and flower visitor richness was the dependent variable. In this model, only flower visitor abundance was related to taxonomic richness (Flower visitor abundance: F_(1,63)_ = 78.06, p<0.0001; Genotypic Diversity: F_(1,63)_ = 0.14, p = 0.71), indicating the higher taxonomic richness of floral visitors observed in genotypically diverse plots was a result of more abundant floral visitors, and not a direct effect of genotypic diversity.

### Non-Additivity

In 2007 and 2008, the effects of genotypic diversity on floral abundance, flower visitor abundance, and flower visitor richness were non-additive at higher levels of genotypic diversity (i.e., the 6 and 12 genotype plots; see Table S1 for full description of null-model results). We use Hughes et al.'s (2008) definition for an additive mechanism as one “for which the ecological response of individual genotypes measured in monoculture, and knowledge of the initial relative abundance of each genotype in a population, are jointly sufficient to predict the same ecological response for a genetically diverse population” [28]. Averaged across 3, 6, and 12 genotype plots, floral abundance was 28% and 11% greater than expected, flower visitor abundance was 40% and 58% greater than expected, and flower visitor richness was 19% and 90% greater than expected in 2007 and 2008, respectively. Expectations were based on results of resampling single genotype plot means.

There are at least two hypotheses that may explain non-additive responses of flower visitors to genotypic diversity: 1) Non-additive flower visitor abundance is a consequence of non-additive plant responses whereby the non-additive plant response is correlated with the non-additive flower visitor response; 2) Non-additive flower visitor abundance is a consequence of intra-guild interactions whereby the non-additive response of flower visitors is independent of the plants. Consistent with the first hypothesis that non-additive plant responses can lead to non-additive responses among flower visitors, when we excluded the single genotype plots where non-additive plant responses cannot occur, we found that there was a strong correlation between the non-additive response (observed value/additive expectation) of plant flowering and non-additive flower visitor abundance (Full Model: F_(2,6)_ = 11.97, p = 0.037, r^2^ = 0.89; Non-additive plant flowering: F_(1,6)_ = 22.98, p = 0.017; Year: F_(1,6)_ = 6.39, p = 0.086).

## Discussion

### Plant Genetics and Floral Communities

Over a two year period, our results provide evidence that both intraspecific genetic variation and genotypic diversity in *S. altissima* indirectly affect flower visitor abundance and richness through their direct effects on floral abundance. These results are some of the first to demonstrate that floral community phenotypes may vary in response to plant genotypic diversity. This represents an important advance as recent studies such as Klein *et al.* (2003) have reported that bee diversity was positively related to fruit set in coffee plantations [31], suggesting that pollinator community dynamics are important to crop plant fitness [31]–[34]. Recent studies also indicate that the genotype of an individual plant can result in an extended phenotype, that is, a phenotype which has extended consequences at the community and ecosystem level [14]. Consistent with the concept of extended phenotypes, our results provide evidence that genetic variation in floral abundance is a mechanism for extended effects on flower visitor abundance and richness (through effects on visitation). Genotypes which had produced more flowers at the time of sampling had a greater number of flower visitors. These genotypes can attract more flower visitors for several reasons, including: [1] an increased availability of nectar and pollen resources [4], [10], and [2] a better foraging/successful feeding tradeoff that can be important to the fitness of flower visitors with short flight ranges [5], [10], [35]. Although many studies have shown that increased floral abundance can attract more flower visitors to a given flower patch [33]–[34], [36] these studies have not been extended to the level of genotypic diversity.

Consistent with the hypothesis that flower visitors are more abundant where plant genotypic diversity is high, our results showed that increased *S. altissima* genotypic diversity was positively related to floral abundance and flower visitor abundance. *S. altissima* patches with high genotypic diversity produced more flowers (i.e., an over-yielding effect, the mechanisms of which are unknown), which made these plots more attractive to flower visitors. This suggests that floral abundance may mediate the positive effect of genotypic diversity on flower visitor abundance. Genotypically diverse plots had greater floral abundance, suggesting that positive genotype interactions occur in mixture plots. One potential explanation for greater floral abundance plots is increased productivity in genotypically diverse plots compared to monocultures, which has been previously shown in the *S. altissima* system [23]. Such indirect genetic effects have recently been shown to be important to non-additive responses at the community and ecosystem level [1].

### Synergistic Non-Additive Effects on Flower Visitors

Non-additive responses occur when the total response is greater or less than the sum of the partitioned responses generated by the individual constituents. Although emerging studies have shown that genotypic interactions are important to non-additive outcomes at the community and ecosystem level, the exact mechanisms which promote non-additive responses are not well understood [16]–[18], [23]–[25]. We found synergistic non-additive responses of floral abundance and flower visitor abundance in genotype mixture plots. Consistent with those findings, we found that non-additive responses of plants were correlated with non-additive responses of flower visitors. Such synergistic, positive outcomes suggest that complementarity in mixture plots may be due to phenotypic plasticity which would enable genotypes to occupy different niches than they would when planted in isolation. Supporting this idea, empirical [4]–[5] and theoretical [37] studies have suggested that different plant species can facilitate each other's pollination. It has also been proposed that individual plants in diverse floral communities have higher pollination rates as a result of different and complementary floral rewards [4], [10]. This implies that the quality and quantity of nectar in neighboring plant species can attract pollinators to each other that may not have been attracted otherwise. However, the exact mechanisms of potentially facilitative interactions for increased flower visitor abundance within genetically diverse patches of *S. altissima* remain unknown.

### Conservation Implications

Because of the general importance of pollinators to associated biodiversity and ecosystem function, understanding the mechanisms of global decline in pollinators and the services they provide represents a major frontier in ecology [3]. Although current hypotheses explaining declines in pollinators include disease, parasites, changing agricultural practices, and habitat fragmentation/destruction, [3], [38]–[39], our results raise an additional hypothesis that declines in plant genetic variation may negatively influence flower visitors by reducing floral resource availability. Evidence that plant genetic factors affect pollinator dynamics in a natural system may have important implications for agricultural systems, as genetic variation in many animal-pollinated crop plants is reduced to few varieties [3]. Reductions in genetic variation in both agricultural and natural systems result in synchronous flowering in plants and a “boom and bust” resource for pollinators. Such a “feast or famine” cycle represents an additional mechanism to explain the ongoing decline in some pollinators. Our preliminary results clearly show that floral visitors were more abundant on plots established with multiple genotypes because these plots produced more flowers. Such results suggest that plant genetic variation may be a powerful tool for maintaining environmental sustainability in natural and agricultural systems. Further research is needed to examine if these effects are consistent at the landscape scale, where plots can be orders of magnitude larger. Importantly, because the effects of genotypic diversity on flower visitor abundance are non-additive, they are suggestive of threshold effects. Although the most recent, comprehensive assessment of the factors affecting pollinator decline addresses the potential role of plant genetic variation in the context of Allee effects leading to extinction in plant populations [3], our results show that plant genetic variation and diversity impact the abundance of floral visitors and suggest that further research on this topic is warranted.

## Materials and Methods

Tall goldenrod (*Solidago altissima* L.) is a dominant species in abandoned agricultural fields, where it can have major impacts on biodiversity and ecosystem function [23], [40]–[41], making it an ideal species to examine how floral visitor communities and ecosystem function vary in response to intraspecific genetic variation. *S. altissima* is a common perennial herbaceous species which is broadly distributed across North America and readily produces clones which can persist for many years [42]. Genetic diversity of natural *S. altissima* patches can vary from 1 to 12 genotypes in less than a meter-square area creating a natural mosaic of single-genotype and mixed-genotype plant patches [42].

*Solidago altissima* is obligately outcrossed and animal pollinated [43] which makes it ideal for understanding how genetic variation in plants affects associated flower visitors. The flowers are pollinated by a diverse community of arthropods including many Hymenoptera, Lepidoptera and Coleoptera [43]. The *S. altissima* inflorescences (capitula) form a panicle at the stem apex and buds open almost synchronously within a particular genotype; however, flowering phenology varies among clones [43]. Moreover, many studies have shown that plant genetic factors associated with *Solidago* spp. influence trophic interactions among galling herbivores and natural enemies, as well as arthropod diversity at large [23], [41], [44]–[49]. Together, high ecological and genetic variation and ease of propagation make *S. altissima* an ideal species to investigate how plant genetic factors influence floral community dynamics, the results of which may be applicable to many other plant species.

We examined the role of plant genetic factors on flower visitors with a common garden experiment established in 2005 in the National Environmental Research Park at Oak Ridge National Laboratory in Oak Ridge, Tennessee. Genotypic diversity of *S. altissima* was manipulated at the plot level by using 21 locally-collected genotypes. The genotypes were identified as unique by amplified fragment length polymorphism (AFLP). In 2005, sixty-three 1 m^2^ plots of equal stem density were established at random locations within the common garden with 12 replicated individuals of 1, 3, 6, or 12 randomly selected genotypes. Each plot was spaced 1m from its nearest neighbor plots. Bamboo posts were placed in each corner of the plots, and string was tied around the posts to prevent plants from different plots from touching each other. Single genotype plots included 2 replicate plots of each of the 21 genotypes (42 total). Genotypically diverse plots included 7 replicates of each of the 3, 6, and 12 genotype mixtures (21 total). The polyculture plots were created by a random assignment of genotypes, with the stipulation that no two mixtures could have exactly the same constituent genotypes. Heavy plastic lined the edges of each plot to a depth of 30 cm to prevent ramets from spreading to neighboring treatments, but ramets were allowed to spread within each plot over time.

To determine whether plots were impacted by their proximity to neighboring plots, we tested for spatial autocorrelation by comparing the abundance of flower visitors in each plot to the average abundance of flower visitors on neighboring plots [50]. Using linear regression, we found no evidence that plots were influenced by their neighbors with respect to the abundance of flower visitors (F_(1,63)_ = 1.23, p = 0.223). Similarly, when genotype identity and flower abundance were included in the model as covariates, we still found that the average number of flower visitors on neighboring plots did not influence the abundance of flower visitors in a focal plot (F_(1,63)_ = 0.27, p = 0.613). In September 2007 and 2008, we surveyed each plot within the common garden to estimate the proportion of flowers in bloom. Because flower panicles vary in size, we used a representative panicle of *S. altissima* with known floral (capitula) abundance as a unit of measurement to estimate floral abundance. For each plot, we visually estimated floral abundance as the number of times the representative panicle would have to be replicated in order to equal the floral abundance of the plot [51], and then counted the number of inflorescences on the representative panicle to obtain an estimate of the total number of inflorescences per plot. We measured the abundance of floral visitors by observing each of the plots for two minutes (with two observers) and recording all visitors to flowers. Common flower visitors include six general taxa: honeybees (Hymenoptera: Apidae), sweat bees (Hymenoptera: Halictidae), bumblebees (Hymenoptera: Apidae), *Polistes* wasps (Hymenoptera: Vespidae), *Ailanthus* webworm moths (Lepidoptera: Yponomeutidae), and skippers (Lepidoptera: Hesperidae). Other taxa of floral visitors were extremely rare, and were not included in our analysis. The abundance of floral visitors was calculated as the number of individuals of each taxa entering the plot during the observation period, regardless of the number of flowers each individual visited within the plot. We used the six general taxa to judge the taxonomic richness of floral visitors visiting each plot. We use the terms “taxonomic richness” and “richness” interchangeably to refer to the number of flower visitor taxonomic groups observed in a given plot. We observed significantly higher floral visitation and richness in 2007 compared to 2008 although overall patterns in the results between 2007 and 2008 were similar.

### Statistical Analyses

To test for differences in phenotypic and extended phenotypic traits (traits which impact levels beyond the individual; see [14]) across 21 different genotypes of *S. altissima*, we used restricted estimated maximum likelihood in SAS-JMP 5.1. The statistical model included plant genotype and row as random effects. The significance of plant genotype on floral abundance, flower visitor abundance, and flower visitor taxonomic richness was tested with a log-likelihood ratio test. Because the genotypes of *S. altissima* used in this study were clonally replicated we calculated the broad-sense heritability for each trait as H^2^ *_B_* = V*_g_*/V*_t_* where V*_g_* is the amount of variation in the trait that is explained by genetic variance and V*_t_* is the total variance in the phenotype of the trait (genetic and environmental). The standard error for H^2^ depends on the intraclass correlation (t), the number of clones (S), and the number of individuals per clone (k). According to Becker (1985), the following formula approximates SE, assuming that t is normally distributed: S.E. = {[2 (n.−1)(1−t)^2^ (1+(k_1_−1) t)^2^]/[k_1_^2^ (n.−S)(S−1)]}^1/2^ [52].

To examine the relationship among plants and flower visitors in this system we used regression analysis with floral abundance as the independent factor and flower visitor abundance and richness as the dependent variables. We used genetic correlations rather than phenotypic correlations to avoid confounding effects of environmentally induced covariance between traits [29], [30].

To understand how genotypic diversity affects *S. altissima* floral abundance and associated flower visitor abundance and richness, we used regression analysis with level of genotypic diversity as the independent variable. All significant relationships showed a decelerating positive response to genotypic diversity, and were fitted using 2-parameter logarithmic best-fit lines; floral abundance in 2008 and flower visitor richness in 2007 were square-root transformed. This test allowed us to determine if the measured traits changed in response to genotypic diversity. Average trait values for each genotypic class (i.e., 1, 3, 6, or 12) were also used as observed values to test for non-additivity against a null model.

Non-additivity occurs if the total response of a variable is greater or less than the sum of the partitioned responses generated by the individual constituents [23]–[25]. Experiments designed to test for the effects of genotypic diversity typically examine non-additivity to determine if changes in the response variable resulted from presence of a particular genotype causing a strong response or caused by an interaction of genotypes that led to a synergistic or antagonistic effect on the response variable. We used Loreau and Hector's (2001) method for calculating complementarity effects by comparing the average relative yield in mixture compared to monoculture [53]. We could not explicitly calculate a selection effect, because we could not visually identify ramets as different genotypes. We created “null communities” based on initial genotype frequencies to test if our observed values for floral abundance, flower visitor abundance, and flower visitor richness richness differed significantly from additive expectations. To generate null communities, we created a list, outlining how many times each genotype appeared in each diversity treatment (i.e. 3, 6, or 12 genotypes). This was done because the diversity plots were created by a random draw of genotypes, and each genotype was not equally represented in each diversity treatment. We resampled one or more monoculture plot mean(s) without replacement for each genotype on the list to recreate a series of null plots for each treatment. We then summed the resampled plot means, divided by the total number of plots sampled, and repeated this process 999 times, so that the process was done a total of 1000 times. This method was repeated for the 6 and 12 genotype plots. We compared our observed treatment mean values to the distribution of null model results, and when our observed value fell within the top or bottom 2.5% of the distribution, the test indicated (at α = 0.05) that there were non-additive effects of genotypic diversity.

We recognize that the genotypic diversity of the plots has likely not remained constant over time, which could affect the accuracy of our null model. However, the effects of genotypic diversity were extremely similar between the 2007 and 2008, suggesting that selection on particular genotypes was not strong during the time we were collecting data. Our data do clearly show a non-additive effect [28], although to what extent this effect is due to selection on particular genotypes relative to complementarity effects among genotypes is unknown.

## Supporting Information

Table S1 This table presents the results of null model simulations testing for non-additivity in floral abundance, floral visitor abunance, and floral visitor richness in 2007 and 2008. Expected values are mean results from null model simulations, and lower and upper confidence intervals (CI) represent 95% confidence intervals. P-values represent the number of simulations expressed as a proportion out of 1000 which fall above the observed value. Non-additivity values represent the percent increase in observed values above expected values.
(0.05 MB DOC)
