# Supplementary material for: Non-Additive Effects of Genotypic Diversity Increase Floral Abundance and Abundance of Floral Visitors
Source: PLoS One. 2010 Jan 14;5(1):e8711. doi: 10.1371/journal.pone.0008711 (PMC2806830; doi:10.1371/journal.pone.0008711)
Supplement: Table S1 — This table presents the results of null model simulations testing for non-additivity in floral abundance, floral visitor abunance, and floral visitor richness in 2007 and 2008. Expected values are mean results from null model simulations, and lower and upper confidence intervals (CI) represent 95% confidence intervals. P-values represent the number of simulations expressed as a proportion out of 1000 which fall above the observed value. Non-additivity values represent the percent increase in observed values above expected values. (0.05 MB DOC) [file pone.0008711.s001.doc]

**Supplementary Table 1. Resampling results and non-additivity.**

| Genotypic Diversity | Variable | Observed | Expected | Lower CI | Upper CI | p | Non-Additivity |
| --- | --- | --- | --- | --- | --- | --- | --- |
| 3 | Floral Abundance (2007) | 1848 | 1733 | 1425 | 2041 | 0.534 | 7% |
| 6 | Floral Abundance (2007) | 1917 | 1975.05 | 1709 | 2125 | < 0.001 | 21% |
| 12 | Floral Abundance (2007) | 1656 | 1655.50 | 1536 | 1771 | < 0.001 | 57% |
| 3 | Floral Vis. Abun. (2007) | 6.29 | 5.32 | 4.57 | 6.10 | 0.006 | 18% |
| 6 | Floral Vis. Abun. (2007) | 8.00 | 5.91 | 5.31 | 6.52 | < 0.001 | 35% |
| 12 | Floral Vis. Abun. (2007) | 8.00 | 4.82 | 4.46 | 5.18 | < 0.001 | 66% |
| 3 | Floral Vis. Richness (2007) | 2.14 | 2.19 | 2.00 | 2.38 | 0.768 | -2% |
| 6 | Floral Vis. Richness (2007) | 2.71 | 2.24 | 2.07 | 2.40 | < 0.001 | 21% |
| 12 | Floral Vis. Richness (2007) | 2.71 | 1.96 | 1.82 | 2.08 | < 0.001 | 38% |
| 3 | Floral Abundance (2008) | 1767 | 1847 | 1661 | 2045 | 0.530 | -4% |
| 6 | Floral Abundance (2008) | 1850 | 1757 | 1583 | 1925 | 0.064 | 5% |
| 12 | Floral Abundance (2008) | 2073 | 1560 | 1406 | 1724 | < 0.001 | 33% |
| 3 | Floral Vis. Abun. (2008) | 2.14 | 1.71 | 1.52 | 1.90 | < 0.001 | 25% |
| 6 | Floral Vis. Abun. (2008) | 3.00 | 1.85 | 1.61 | 2.10 | < 0.001 | 62% |
| 12 | Floral Vis. Abun. (2008) | 2.71 | 1.45 | 1.36 | 1.55 | < 0.001 | 87% |
| 3 | Floral Vis. Richness (2008) | 0.86 | 0.45 | 0.38 | 0.52 | < 0.001 | 90% |
| 6 | Floral Vis. Richness (2008) | 0.86 | 0.57 | 0.48 | 0.67 | < 0.001 | 50% |
| 12 | Floral Vis. Richness (2008) | 1.14 | 0.49 | 0.44 | 0.54 | < 0.001 | 131% |
